# Modified coracoid tunnel-free suspended bridge for coracoclavicular ligament injuries leads to improved clinical and radiological outcomes: a cohort study with a minimum 2-year follow-up

**DOI:** 10.1186/s12893-026-03547-2

**Published:** 2026-02-07

**Authors:** Gang Liu, Lin Li, Shitian T. Tang

**Affiliations:** Department of Orthopaedics, School of Medicine, Mianyang Central Hospital, University of Electronic Science and Technology of China, No.12 Changjia Lane, Fucheng District, Mianyang, 621000 Sichuan Province PR China

**Keywords:** Acromioclavicular joint, Dislocation, Coracoclavicular ligament, Arthroscopy, Suspended bridge, Complication

## Abstract

**Background:**

Coracoclavicular ligament injuries are common in the sports population. Coracoid tunnel techniques showed a negative influence on outcomes with some literature reporting tunnel-related fractures occurring early or late after surgery. The aim of this study is to analyze the clinical and radiological outcomes of modified coracoid tunnel-free suspended bridge in coracoclavicular ligament injury patients with a minimum follow-up of 2 years.

**Methods:**

This retrospective study reviewed patients with coracoclavicular ligament injuries who were treated with modified coracoid tunnel-free suspended bridge between January 2019 and February 2023 at the authors’ institution. Clinical outcomes were patient-reported subjective and objective functional scores, including Visual Analogue Score (VAS), Oxford Shoulder Score (OSS) and Constant-Murley Score (CMS). Radiological assessments were performed immediately after surgery, as well as at 1 month, 3 months, 6 months, 1 year postoperatively, and at the final follow-up. The radiological complications were recorded at final follow-up, including loss of AC (acromioclavicular) joint reduction, recurrent dislocation of AC joint, tunnel widening, or tunnel-related fractures.

**Results:**

A total of 34 patients who were treated with modified coracoid tunnel-free suspended bridge technique were included in the study. All patients completed the final follow-up, with an average follow-up of 30.9 ± 6.8 months. At the final follow-up, these patients achieved good to excellent functional outcomes with the mean Oxford Shoulder score of 13.9 ± 1.9 points and the mean Constant Murley score of 89.7 ± 6.2 points. Two of 34 patients (5.9%) had a loss of AC joint reduction at the final follow-up, but did not report AC joint pain or instability. AC joint showed a trend of secondary displacement within 3 months after surgery. The side-to-side difference of coracoclavicular distance significantly increased from 0.2 mm one month after surgery to 1.6 mm three months after surgery (*p* = 0.004). However, no tunnel-related complications were observed and no patient required revision.

**Conclusions:**

The modified coracoid tunnel-free suspended bridge is a safe procedure that can significantly improve clinical and radiological outcomes of coracoclavicular ligament injuries. This technique could be considered a reliable method for acromioclavicular joint reconstruction.

## Background

Acromioclavicular (AC) joint separation is one of the most common shoulder joint injuries in the sports population. High-grade AC joint dislocation is usually treated surgically due to the coracocavicular (CC) ligament and AC ligament injuries. In most surgical techniques, the use of coracoid tunnel technique for CC ligament reconstruction is more popular than other techniques [1–3]. However, the risk of tunnel-related fractures occurring early or late after surgery has been widely reported, with incidence rates ranging from 2.4% to 5.7% [4–8]. Most patients with tunnel-related fractures end up receiving surgical treatment due to shoulder pain, AC joint instability, and activity-related weakness [7].

There is no consensus on the optimal surgical technique for CC ligament injuries. Milewski et al. [9] reported that a variety of complications can occur, including clavicle fracture and coracoid process fractures via coracoid tunnel techniques. Notably, the coracoid tunnel technique is associated with a particularly high tunnel-related complication rates. Additionally, the precise drilling of coracoid bone tunnel demand extensive surgical experience. At present, the establishment of the bone tunnel is still challenging for some orthopedics departments, especially that of basic-level hospitals, which limits the application of this technology [10]. Therefore, a new less time-consuming technique requiring fewer skills to drill the bone tunnel is needed.

In our series, modified coracoid tunnel-free suspended bridge using the AC Rigidloop System (DePuy, Mitek Inc, Warsaw, IN, USA) and Suture tape (Arthrex Inc, Naples, FL, USA) was used for patients with acute CC ligament injuries. The aim of this study is to evaluate this modified technique towards a patient’s recovery with respect to clinical and radiological outcomes. Our hypothesis was that it may have potential advantages including a simple, fast, small-sized clavicle tunnel and low drilling-related fracture. We assume that this technique can significantly improve clinical and radiological outcomes, and can be widely promoted in basic-level hospitals. These findings will potentially guide surgeons toward more effective treatment strategies for CC ligament injuries.

## Methods

### Ethics statement

This study was approved by the Ethical Committee of the authors’ affiliated institutions (2023KY032). All patients signed written informed consent forms and agreed to publish their images for medical research.

### Study design and participants

The clinical data of all patients with acute CC ligament injuries treated with modified coracoid tunnel-free suspended bridge at our institute from January 2019 to February 2023 were retrospectively reviewed. The inclusion criteria were as follows: (1) adult patients under 70 years of age. (2) diagnosis with dislocation of the AC joint or distal clavicle fracture secondary to acute CC ligament injuries requiring surgery. (3) acute lesions within 3 weeks. (4) a minimal clinical and radiological follow-up of 2 years. Patients who met one or more of the following criteria were excluded: (1) Rockwood type I or II AC joint dislocation, (2) coracoid fracture, (3) the fracture of the distal clavicle is confined to a region located more than 1.5 cm proximal to the AC joint, (4) chronic AC joint dislocation (≥ 3 weeks), (5) open injury, (6) neurological disorder, (7) previous shoulder surgery, (8) incomplete follow-up data or loss to follow-up.

### Surgical technique

**Fig. 1:**
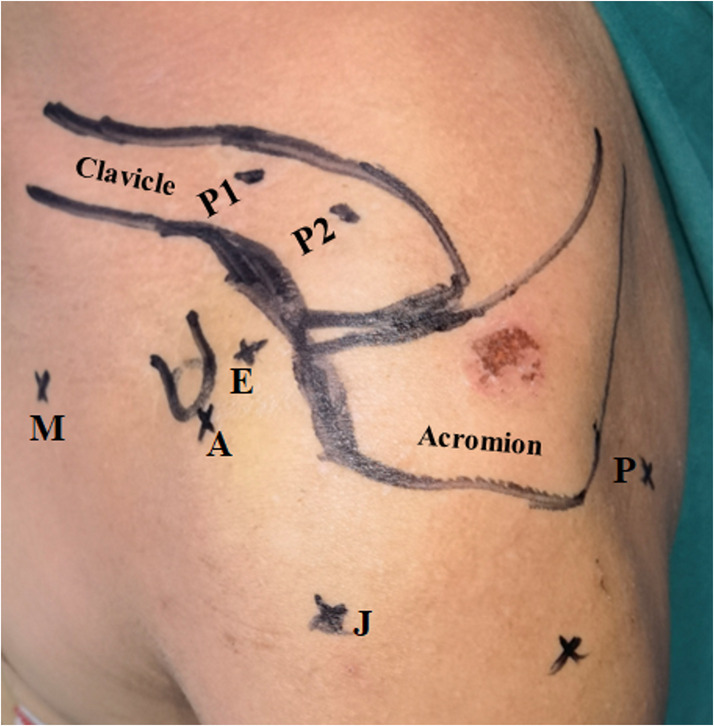
Surgical approach: the K-wire position under C-arm guidance, the medial bone tunnel (P1) and lateral bone tunnel (P2). Surgical portals included the ‌standard posterior P portal for direct visualization of intra-articular pathology, the ‌anterolateral J portal to facilitate arthroscopic view of the coracoid process, the ‌anterior A portal for the soft tissue debridement, and the M working portal (5-o’clock portal), which was located at 4 cm to 6 cm medial to the anterior axillary fold perpendicular to the nipple

The surgery was performed by one surgeon under general anesthesia in a beach-chair position. First, two 1.5 mm guide pins were inserted through the skin according the positions of the P1 medial bone tunnel and P2 lateral bone tunnel on the clavicle (Fig. 1). The positions of these bone tunnels were created via X-ray to ensure adjacent insertion of the conoid ligament and trapezoid ligament as much as possible. Next, diagnostic arthroscopy was performed through a standard posterior P portal and anteromedial E portal (Fig. 1) to explore any accompanying hidden intra-articular lesions. Open the rotator cuff interval until the coracoid process and conjoined tendon could be clearly visualized using posterior P portal, and the camera was placed in the anterolateral J portal (Fig. 1) in order to accommodate visualization of the medial aspect of the coracoid process. Moreover, the AC joint, distal clavicle, and remnant CC ligament should be examined and debrided with the help of the anterior A portal (Fig. 1) if present, while preserving all the residual CC ligament.

**Fig. 2:**
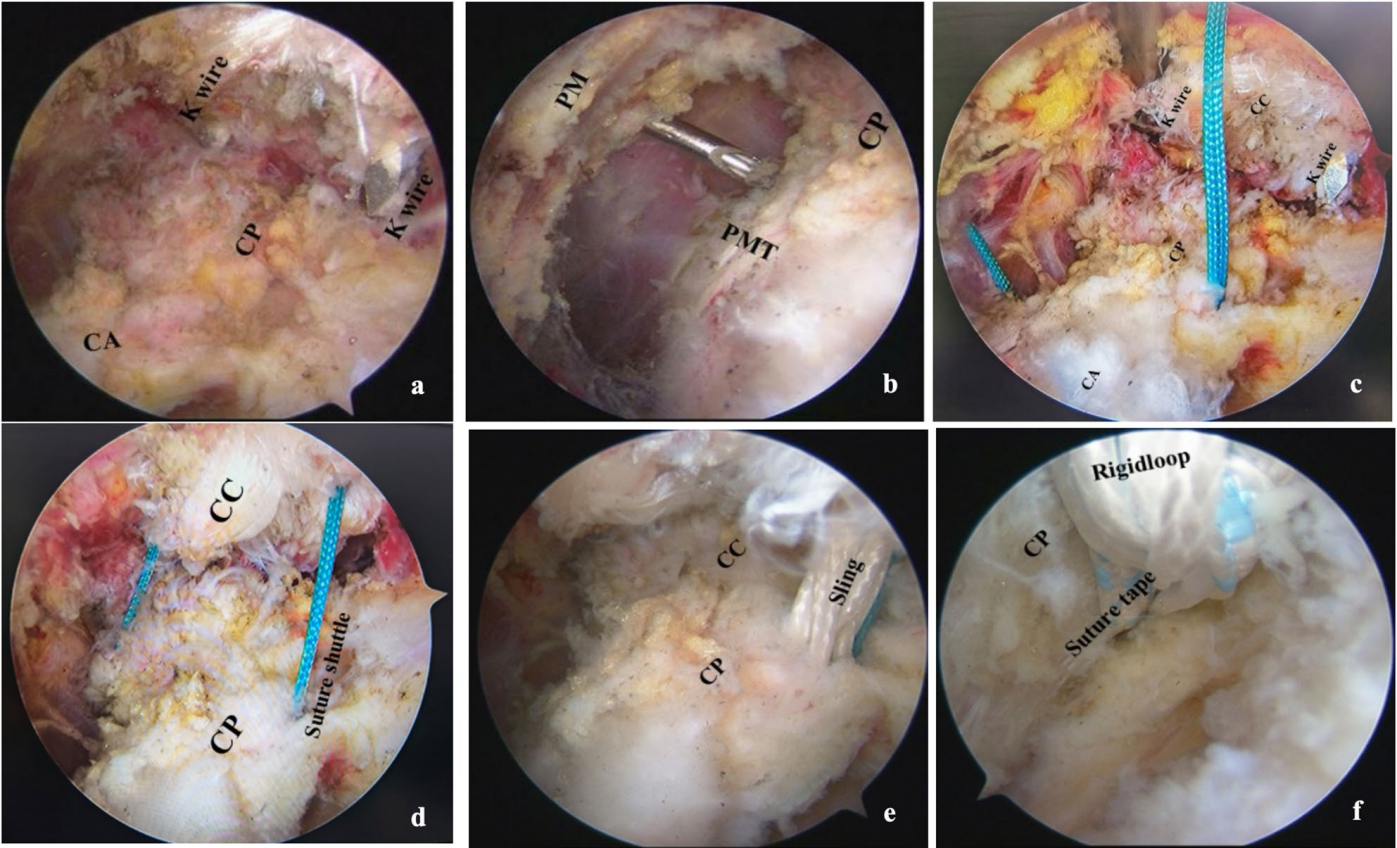
Arthroscopic view of the creation process of bone tunnels and shuttle traction suture. **a** K-wires exploration under arthroscopy via the ‌anterolateral J portal. **b** The partial releasing of the PMT via ‌anterior A portal. **c** A high-strength suture placement under the base of CP through M and E working portals. **d** The prepare of shuttle traction suture using high-strength suture via P1 and P2 bone tunnels of clavicle. **e** Arthroscopic view of the CC space after tightening the Rigidloop system to confirm proper reduction. **f** The position of the Suture tape crossing the base of CP. Note. CP, Coracoid process; CC, Coracoclavicular ligament; PM, Pectoralis minor muscle; PMT, Pectoralis minor tendon; CA, Coracoacromial ligament

The surgeon explored two K-wires along the clavicle under arthroscopy (Fig. 2a). To protect the brachial plexus, the medial aspect of the coracoid process was exposed by partial releasing the pectoralis minor tendon without cutting off (Fig. 2b). Afterwards, the lateral (P2) and medial (P1) clavicle bone tunnels were prepared using a 2.5 mm drill and introduced polydimethylsiloxane synthesis (PDS) traction suture using a spinal needle (forming a loop) via the P2 and P1 bone tunnels respectively (Fig. 1). Then, suture passer loaded with a high-strength suture (forming a loop) was passed underneath the base of the coracoid process (Fig. 2c) via M working portal (Fig. 1), and pulled it to the lateral (P2) bone tunnel using lateral PDS traction suture. Similarly, the contralateral side of high-strength suture was pulled to medial (P1) clavicle tunnels. Consequently, the shuttle traction suture was prepared and passed underneath the base of coracoid process and two bone tunnels of clavicle (Fig. 2d).

**Fig. 3:**
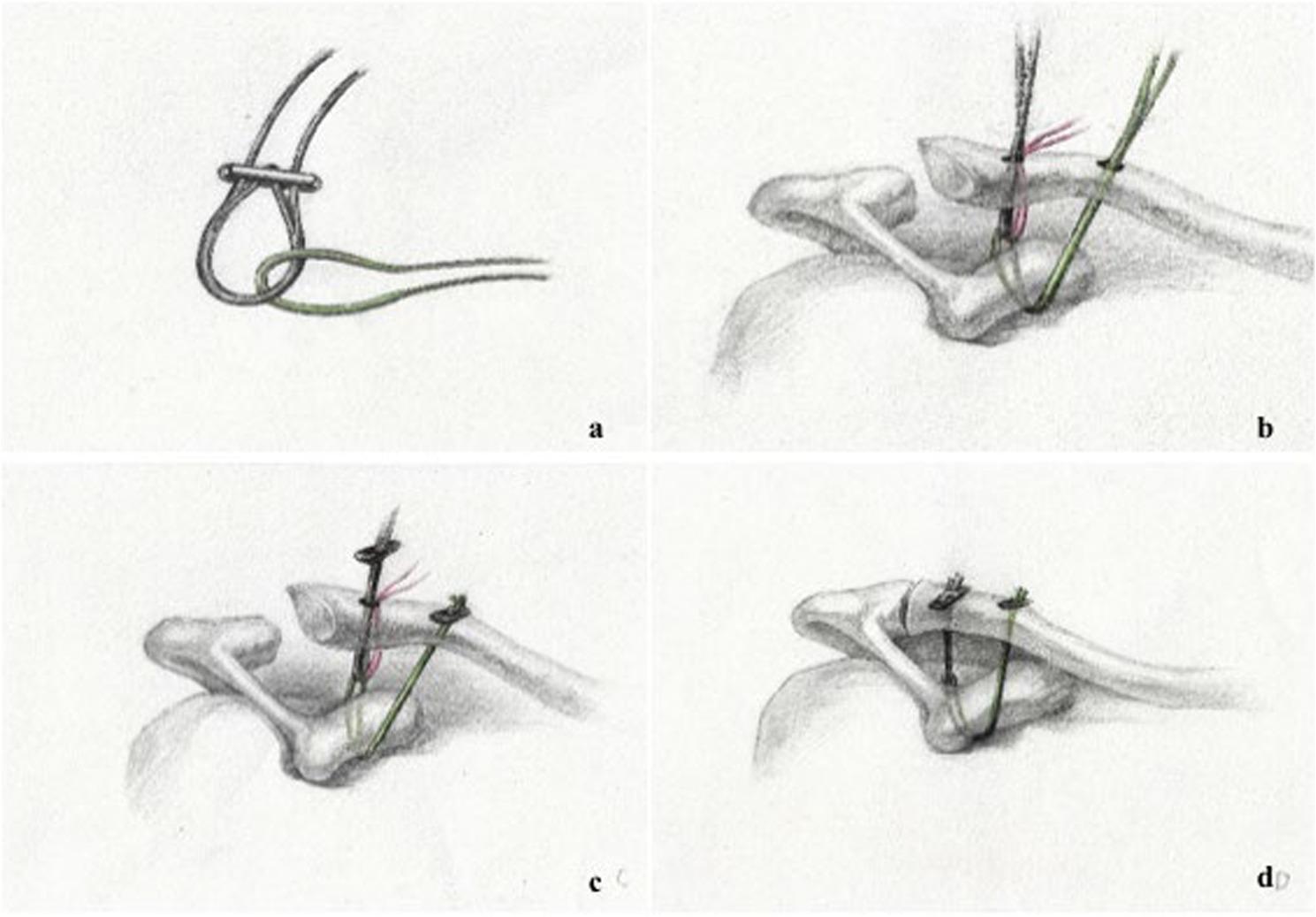
The fabrication of the suspension bridge system and the AC joint reduction and fixation process. **a** The integration of the AC Rigidloop System (DePuy, Mitek Inc, USA) and Suture tape (Arthrex Inc, Naples, FL, USA). **b** The Suture tape is pulled out and passed underneath the base of CP from the P1 tunnel to the P2 tunnel of clavicle using shuttle suture, and its position was adjusted via the pink high-strength suture. **c** A Endobutton plate was used to fix using Suture tape. **d** The AC joint reduction after tightening AC Rigidloop System

A 2.0 mm high-strength Suture tape (Arthrex Inc, Naples, FL, USA) has been passed through the suture loop of Rigidloop system (DePuy, Mitek Inc, Warsaw, IN, USA) (Fig. 3a). Then, a pink high-strength suture was used as traction suture and passed through the Suture tape, as shown in Fig. 3b. Next, the Suture tape was pulled out and passed underneath the base of coracoid process from the P2 tunnel to the P1 tunnel using the shuttle suture (Figs. 2e and 3b). A free Endobutton plate was securely knotted with the Suture tape using a knot pusher (Fig. 3c). During the surgical procedure, the position of the junction of the AC Rigidloop System and the Suture tape must be extended slightly across the base of coracoid process via the pink high-strength suture, and not go too far to avoid limited reduction of the AC joint (Figs. 2f and 3c). When the loop was pulled up, the “sling effect” of AC joint proper reduction can be observed (Fig. 3d). Lastly, X-ray confirmed AC joint dislocation has been completely reduced.

### Rehabilitation protocol

Patients were placed in a simple sling for a period of 4 weeks. After the immobilization, passive activity of shoulder was unrestricted, but active activity was prohibited within 3 to 6 months after surgery. Return to contact sport was generally allowed 9 to 12 months after surgery.

### Clinical evaluation

At final follow-up, functional outcomes were evaluated by an independent surgeon, including the visual analog scale (VAS) pain score, Oxford Shoulder score (OSS) and Constant-Murley score (CMS) [11, 12]. Patient was considered to have excellent result if OSS was 12 to 15 points, good 16 to 30 points, fair 31 to 45 points and poor > 45 points. Additionally, CMS was assessed, and CM score ≥ 90 points was defined as excellent functional result, good 80 to 89 points, fair 60 to 79 points, and poor < 60 points.

### Radiological evaluation

All selected patients were evaluated by non weight-bearing bilateral clavicle AP X-ray immediately, 1 month, 3 months, 6 months, 1 year after surgery, and in the last follow-up. The AC joint separation was classified according to Rockwood type. The coracoclavicular distance (CCD) was defined as the vertical distance of the adjacent cortex between the coracoid and the distal clavicle using X-ray. The acromioclavicular distance (ACD) was defined as the horizontal distance between the anterior edge of the acromion and the lateral clavicle using CT scans. In addition, the status of AC joint was defined as maintained reduction, loss of reduction and complete dislocation. Loss of reduction was defined as a 25% increase in the AC joint width both in the vertical and horizontal plane. Radiological failure was defined as the persistent presence of type III, IV or V AC joint dislocation at the final follow-up.

### Statistical analysis

Statistical analyses were performed using SPSS 25.0 software (IBM Corp, Armonk, NY). Quantitative data were expressed as mean ± standard deviation, and the independent sample *t* test was adopted for comparison. Categorical data were expressed as percentages. The change trend of the radiographic data over time after surgery was also analyzed by one-way ANOVA. All statistical tests were a two-tailed, and significance was set at *P* < 0.05.

## Results

### Patients characteristics

**Fig. 4:**
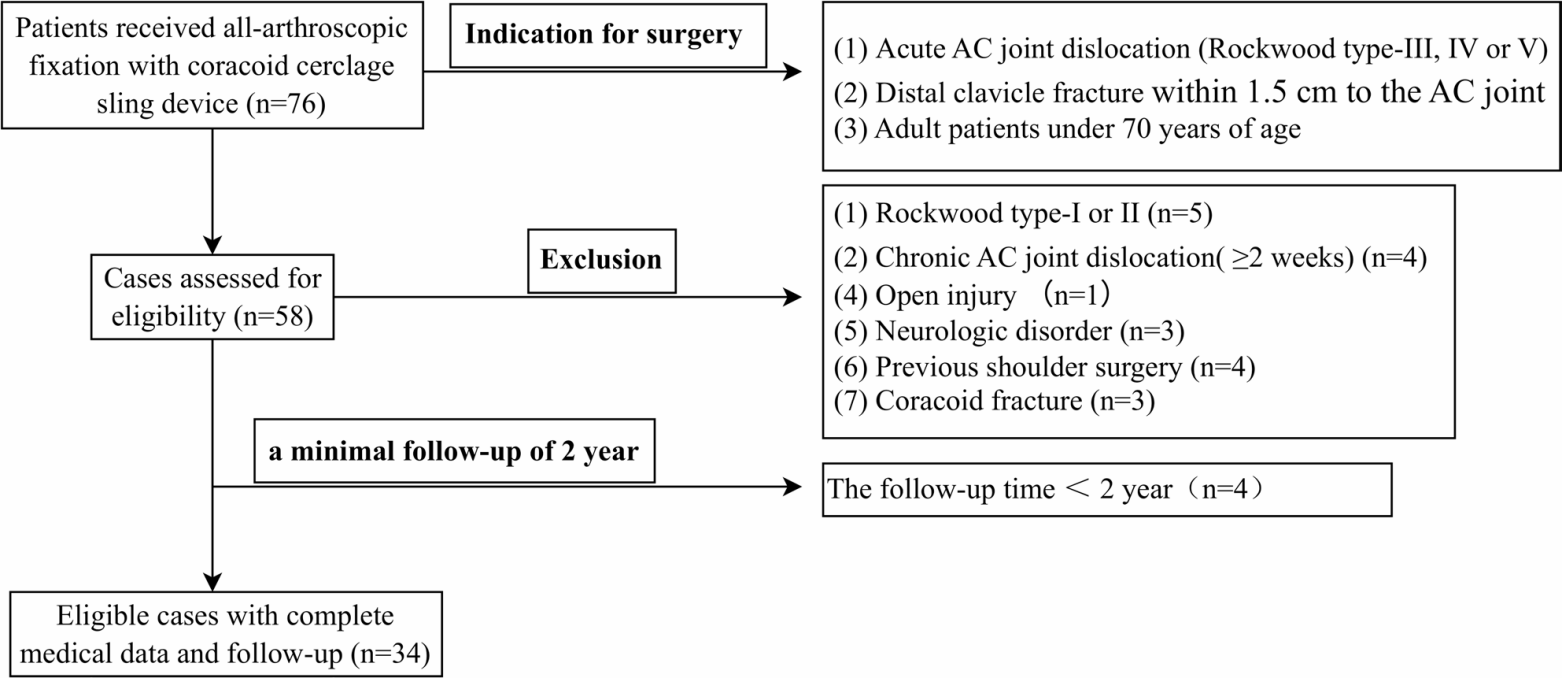
Patient selection in this study (Note. AC joint, acromioclavicular joint)

A total of 34 patients with acute CC ligament injuries underwent total arthroscopic reconstruction using the modified coracoid tunnel-free coracoclavicular suspended bridge technique (Fig. 4).

As shown in Table 1, this study consisted of 24 males (70.6%) and 10 females (29.4%). The mean age was 46 ± 10 year, and the body mass index (BMI) is 22.7 ± 2.6 kg/m^2^. The most common injury mechanism was fall. The dominant side was involved in 28 of the 34 patients (82.4%). Among these 34 patients, 12 (35.3%) had Rockwood type-III injuries and 3 (8.8%) had type-IV injuries, 13 (38.2%) had type-V injuries and 6 (17.7%) had distal clavicle fracture.

**Table 1:** Demographic data

	Study cohort (*n* = 34)
Age (yr.)†	46 ± 10
Sex, n, male/female	24/10
BMI (kg/m2)†	22.7 ± 2.6
Dominant arm	82.4% (28/34)
Rockwood, no.(%)	
III	12 (35.3%)
IV	3 (8.8%)
V	13 (38.2%)
Distal clavicle fracture	6 (17.7%)
Injury mechanism	
Fall	16 (47.1%)
Traffic accident	12 (35.3%)
Sports	6 (17.7%)
Concomitant injuries	
SLAP lesion	4 (11.8%)
Rotator cuff injury	2 (5.9%)
Time from injury to surgery, days †	4.9 ± 5.4
Duration of the surgery, minutes †	60.9 ± 20.0
Length of hospital stay, days †	6.0 ± 1.4

*CC* Coracoclavicular.† The values are given as the mean and standard deviation. *BMI* body mass index

### Clinical outcomes

All patients completed the final follow-up, with an average follow-up of 30.9 ± 6.8 months. The VAS was 1.1 ± 1.0 points. The mean Oxford Shoulder score was 13.9 ± 1.9 points, excellent for 28 patients (82.4%) and good for 6 patients (17.7%). The mean Constant Murley score was 89.7 ± 6.2 points, rated as excellent for 18 patients (52.9%) and good for 16 (47.1%). At the final follow-up, 31 of 34 patients (91.2%) returned to their working levels, and the other 3 patients experienced discomfort in the AC joint when lying on the affected side. One patient underwent debridement 3 months after surgery due to incision infection, however, the incision healed and the patient had normal shoulder function ultimately.

### Radiologic assessment

**Fig. 5:**
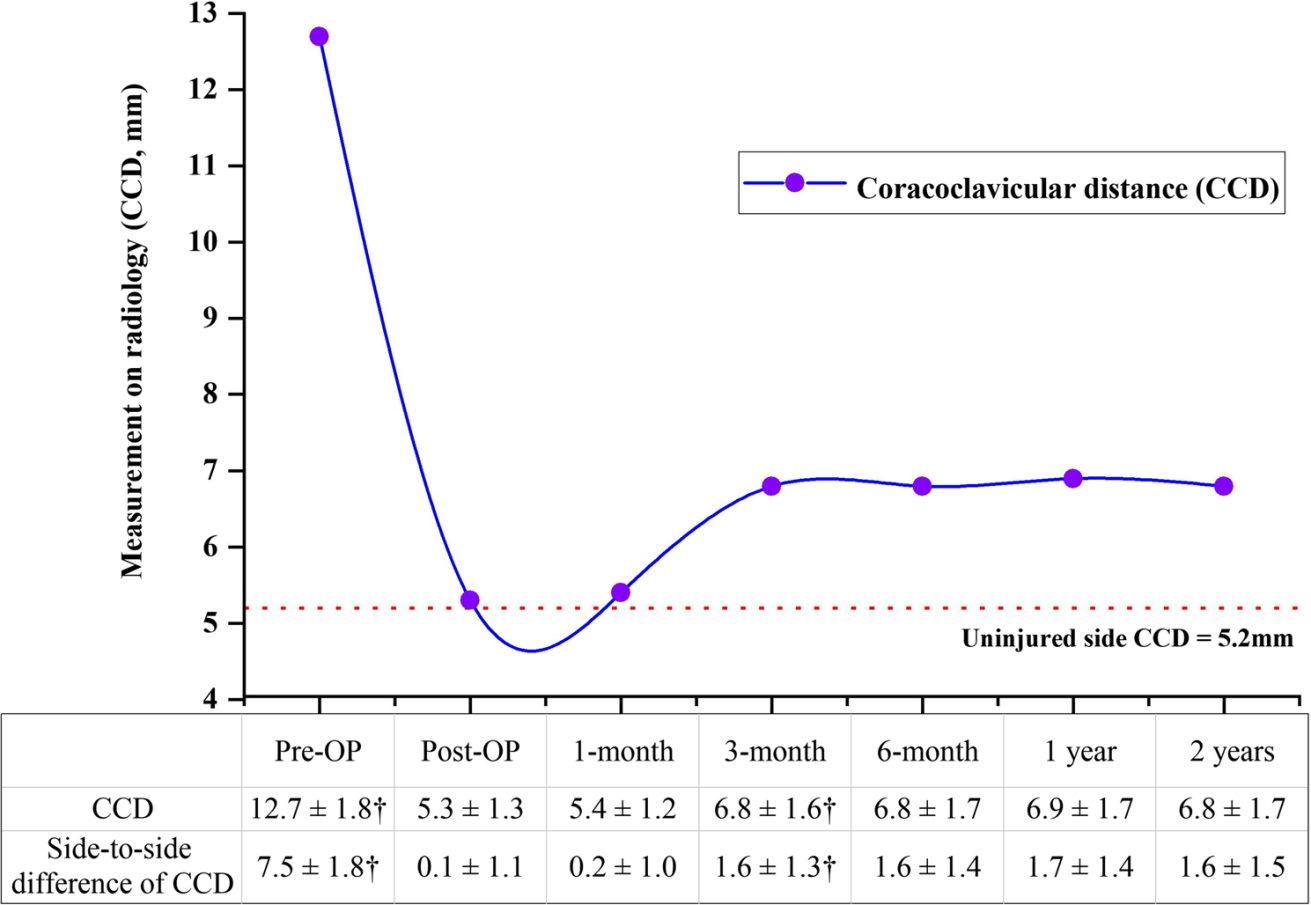
The changes of the CCD in different time points, and kept slight widening over time from 3 months to 6 months postoperatively. (Note. CCD, coracoclavicular distance. †One-way ANOVA)

The changes in CCD and the side-to-side difference of CCD at different time points are shown in Fig. 5. The side-to-side difference of CCD significantly decreased from 7.5 ± 1.8 mm before surgery to 0.1 ± 1.1 mm immediately after surgery (*p* < 0.001). Especially, the surgical treatment of AC joints kept a significant loss of reduction from 0.2 ± 1.0 mm at 1 month to 1.6 ± 1.3 mm at 3 months after surgery (*p* = 0.004). The ACD of 4 patients with AC joint dislocation was 3.2 mm, 3.1 mm, 3.2 mm and 3.6 mm, with the side-to-side difference of 0.8 mm (25.0%), 0.7 mm (22.6%), 0.8 mm (25.0%) and 1.2 mm (33.3%), respectively.

**Fig. 6:**
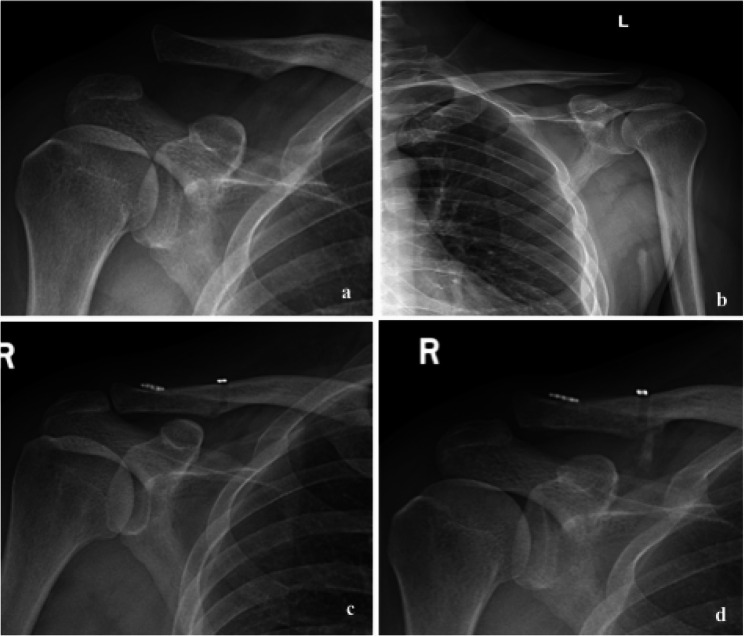
**a** A 46-year-old man with Rockwood type V AC joint dislocation. **b** X-ray film showed healthy X-ray film. **c** X-ray film showed a good reduction of the AC joint after surgery. **d** The operatively treated AC joint was slightly secondary displacement trend at 3 months postoperatively, and calcification of coracoclavicular ligament was observed

**Fig. 7:**
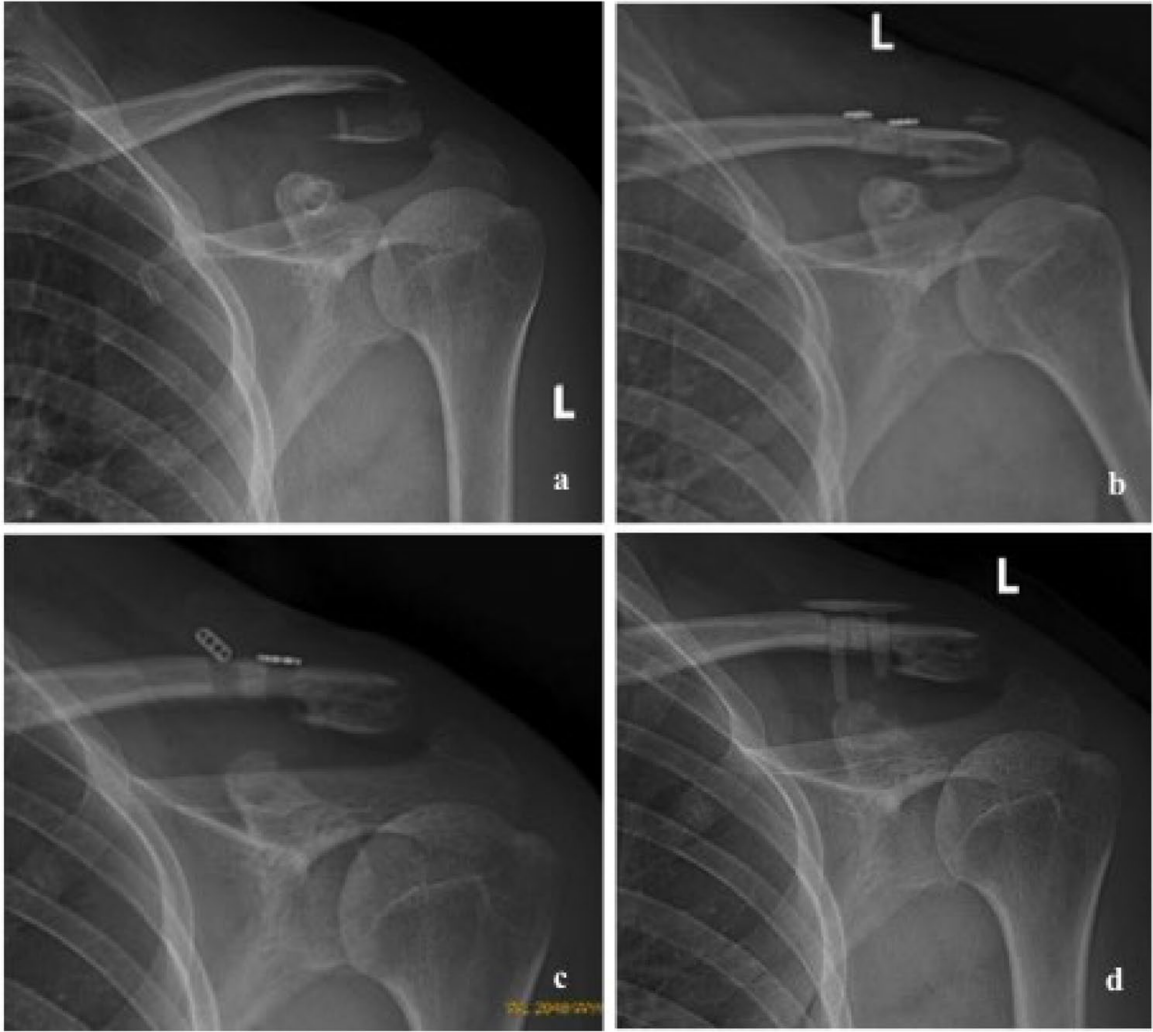
**a** A 31-year-old men with left distal clavicle fracture. **b** X-ray film showed a good reduction of clavicle fracture after surgery. **c** X-ray film showed fixation failure of medial button plate after 2month postoperatively. **d** It had healed completely by use of hardware removal and antibiotic spacer implantation at 3 months postoperatively

At the final follow-up, no patient required revision due to radiological failure. Among these 34 patients, 32 patients (94.1%) maintained complete reduction, two patients (5.9%) had a complete dislocation, and four patients (11.8%) had CC ligament calcification (Fig. 6), but no AC joint pain or instability was reported. In addition, one patient underwent hardware removal and antibiotic spacer implantation due to incision infection 3 months after surgery (Fig. 7). No tunnel-related complications were observed, such as clavicle fractures, coracoid fractures, or tunnel widening.

## Discussion

This study showed that patients with CC ligament injuries obtained satisfactory clinical functional scores and most of the patients returned to their pre-injury working levels after surgery. Additionally, no AC joint pain or instability was reported, and no patient required revision due to radiological failure at the final follow-up. The results revealed the modified coracoid tunnel-free suspended bridge is a reliable method for CC ligament reconstruction.

In our study, the coracoid tunnel-free suspended bridge technique had four advantages: (1) Coracoid tunnel-free technique can reduce the risk of drilling related fracture and iatrogenic neurovascular injuries. (2) The small clavicle tunnels and sutures help to minimize the risk of radiological complications. (3) The technique requires fewer skills and specialized tools. (4) Most importantly, it requires less operational steps. Therefore, it can widely promote the application of this technique in basic-level hospitals.

Recently, there has been an increasing use of Endobutton technique that required a coracoid tunnel for arthroscopic fixation and for the treatment of various AC joint injuries [13–16]. However, this technique often makes it difficult to drill bone tunnels in coracoid, and increases the risk of fractures, button slippage, bone erosion, neurovascular injury, or AC joint instability due to inaccurate placement of button plate in coracoid [17]. Although the early functional score is high, there are also reports of radiological complications related to drilling tunnels [4, 5].

Clavert et al. recently reported that a 22.4% overall complication rate was observed in 116 patients with arthroscopic assisted AC joint reconstruction. Among these patients, 48 (41.3%) patients had persistent dislocation > 150%, and could not return to the same level of movement due to persistent pain [5]. Similarly, in the systematic review, an analysis of 58 articles consisting of 1704 patients showed that compared with open techniques, arthroscopic technique did not reduce the risk of complications. Among these surgical techniques, the hardware failure rate of AC Tightrope technique that requires coracoid tunnel is significantly higher, varying between 4.6% and 17.5% [18]. In fact, although these complications did not show statistically significant differences in functional scores, subjective evaluation and aesthetic subjective satisfaction values were significantly impaired [7, 19]. In contrast, coracoid tunnel-free technique did not require drilling tunnel, which could significantly reduce the tunnel-related complications [17]. To our knowledge, only two studies described clinical outcomes of CC ligament reconstruction via the coracoid tunnel-free suspended bridge using AC Tightrope system [10, 20]. Peng et al. reported that compared with traditional single coracoid tunnel technique, the majority of the patients achieved better clinical results and lower complications [10]. The analysis of 48 cases followed up for 2 to 5 years showed that the tunnel-free coracoclavicular sling technique could significantly improve clinical outcomes without any coracoid drilling-related complications [20]. Similarly, a good to excellent functional state was achieved 2 years after surgery in our series. Most importantly, most patients did not observe any serious radiological complications in this series. However, at 3 months after surgery, the affected AC joint experienced significant secondary loss of reduction over time. At this point, the ligament is usually not fully healed, and repeated stress stimulation may increase the risk of loss of reduction. We believe that the displacement process of the AC joint will ultimately have a good effect on the recovery of shoulder function in long-term follow-up.

In our case series, the 2.5 mm small clavicle tunnel helps to minimize the risk of clavicle fracture, particularly in our cases where the button plate does not pass through the clavicle tunnel. In previous studies, the use of coracoid sling technique with larger clavicle tunnel diameters ranging from 4.0 mm to 7.0 mm was theoretically associated with a higher risk of clavicle fracture and bone erosion [9, 10, 20]. In addition, Thangaraju et al. reported additional widening of bone tunnels. Over time, the gradual widening of the tunnel increases the risk of tunnel-related fractures even 2 years after surgery in a high-risk population [8]. A cadaveric biomechanical study by Spiegl et al. also demonstrated that large bone tunnels may make patients more prone to clavicle fractures after CC ligament reconstruction [21]. In fact, most researchers have reached a consensus that smaller tunnel sizes can significantly reduce drilling related risks compared with larger tunnels [22–24].

In addition, the mechanical properties of different sling materials may be the main reason for the loss of AC joint reduction [25, 26]. Choi et al. found that allograft tendons cannot provide stronger stability due to the gradual elongation of the graft [25]. Some studies also observed 47% of reduction losses and 20% of complications after CC ligament reconstruction with an autologous tendon graft, which adversely affected the results [26]. Besides, due to the sawing effect of sutures through the coracoid, the use of slender Orthocord suture may lead to progressive bone erosion [27, 28]. However, Suture tape cerclage system was a suitable augmented supplement to CC ligament reconstruction and provide significantly greater load and decreased displacement under cyclic loading [29]. Therefore, this technique represents better mechanical performance and minimizes the sawing effect of sutures. Moreover, it requires fewer skills and special tools. Most importantly, it requires less operational steps. Thus, it can widely promote the application of this technique in basic-level hospitals.

### Limitations

The findings should be interpreted with consideration of the following several limitations, including a small sample size and non-comparative design, and inevitable selection bias. Moreover, it is necessary to observe the potential risk of fractures over a relatively long follow-up period. Finally, more accurate and repeatable positioning needs to be studied in future, because the malposition of bone tunnel may lead to early failure [30, 31].

## Conclusion

In conclusion, the presented study proved that application of modified coracoid tunnel-free suspended bridge technique achieved better clinical outcomes with fewer complications in coracoclavicular ligament injuries. This technique could be considered a reliable method for coracoclavicular ligament reconstruction.

## Data Availability

The data that support the findings of this study are available on request from the corresponding author.

## Abbreviations

PDS: Polydimethylsiloxane synthesis
CA: Coracoacromial
CC: Coracoclavicular
VAS: Visual analog scale
OSS: Oxford Shoulder score
CMS: Constant-Murley score
CCD: Coracoclavicular distance
ACD: Acromioclavicular distance
